# Supplementary material for: Click Chemistry of Melamine Dendrimers: Comparison of “Click-and-Grow” and “Grow-Then-Click” Strategies Using a Divergent Route to Diversity
Source: Molecules. 2022 Dec 23;28(1):131. doi: 10.3390/molecules28010131 (PMC9822332; doi:10.3390/molecules28010131)

Supporting Information

Click Chemistry of Melamine Dendrimers: Comparison of "Click-and-Grow" and "Grow-then-Click" Strategies Using a Divergent Route to Diversity

Sanami Numai [1], Risako Yoto [1], Masataka Kimura [1], Eric E. Simanek [2], and Yoshikazu Kitano [1,*]

[1] Laboratory of Bio-organic Chemistry, Tokyo University of Agriculture and Technology, 3-5-8 Saiwai-cho, Fuchu-shi, Tokyo 183-8509, Japan, e-mail: kitayo@cc.tuat.ac.jp; Tel.: +81-42-367-5700

[2] Department of Chemistry & Biochemistry, Texas Christian University, Fort Worth, TX 76129, USA

Table of Contents | **Page**

1.	^{1}H and ^{13}C NMR spectral data of G1 dendrimer **3**	S2
2.	^{1}H and ^{13}C NMR spectral data of G1 dendrimer **4**	S3
3.	^{1}H and ^{13}C NMR spectral data of G1 dendrimer **5**	S4
4.	^{1}H and ^{13}C NMR spectral data of G2 dendrimer **6**	S5
5.	^{1}H and ^{13}C NMR spectral data of G2 dendrimer **7**	S6
6.	^{1}H and ^{13}C NMR spectral data of G2 dendrimer **8**	S7
7.	^{1}H and ^{13}C NMR spectral data of G3 dendrimer **9**	S8
8.	^{1}H and ^{13}C NMR spectral data of G3 dendrimer **10**	S9
9.	^{1}H and ^{13}C NMR spectral data of G1 dendrimer **11**	S10
10.	^{1}H and ^{13}C NMR spectral data of G2 dendrimer **12**	S11
11.	^{1}H and ^{13}C NMR spectral data of G2 dendrimer **13**	S12
12.	^{1}H and ^{13}C NMR spectral data of **15**	S13
13.	^{1}H and ^{13}C NMR spectral data of **16**	S14
14.	^{1}H and ^{13}C NMR spectral data of **17**	S15
15.	^{1}H and ^{13}C NMR spectral data of G3 dendrimer **18**	S16

G1 dendrimer **3**

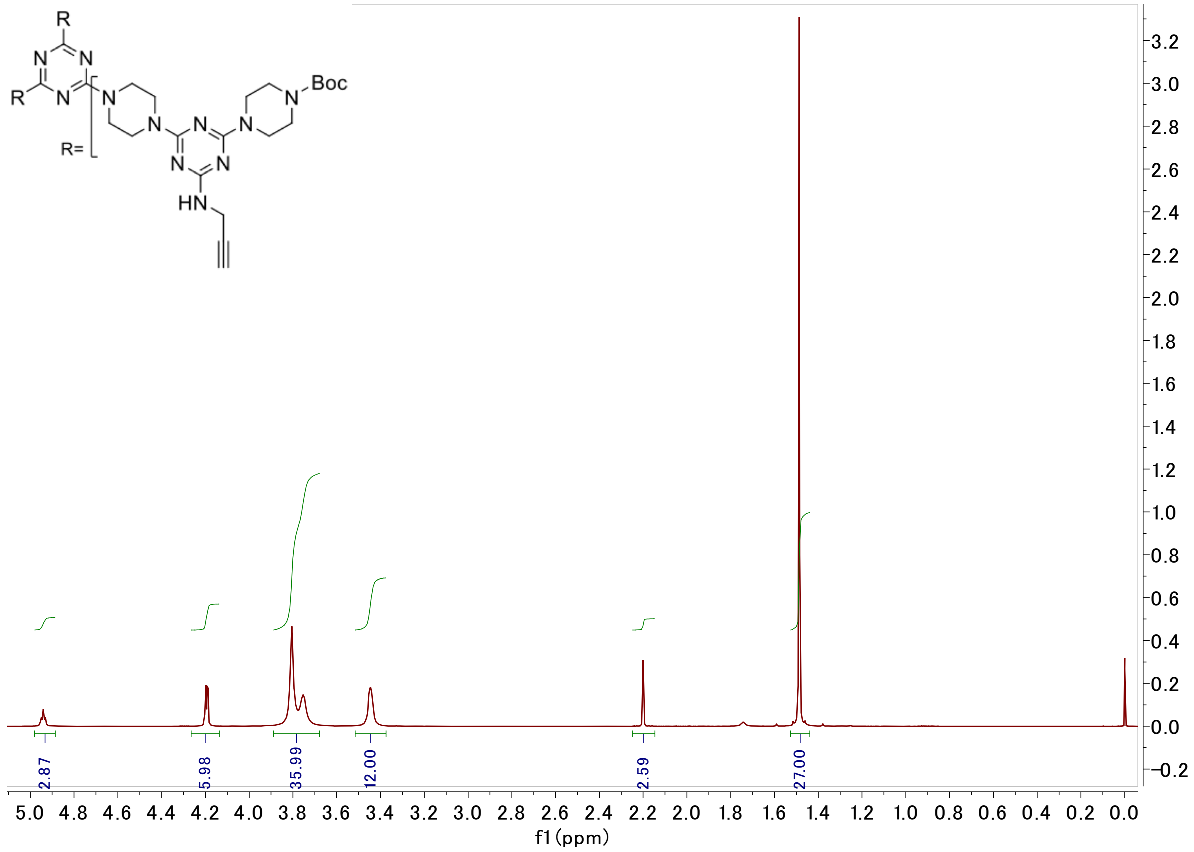

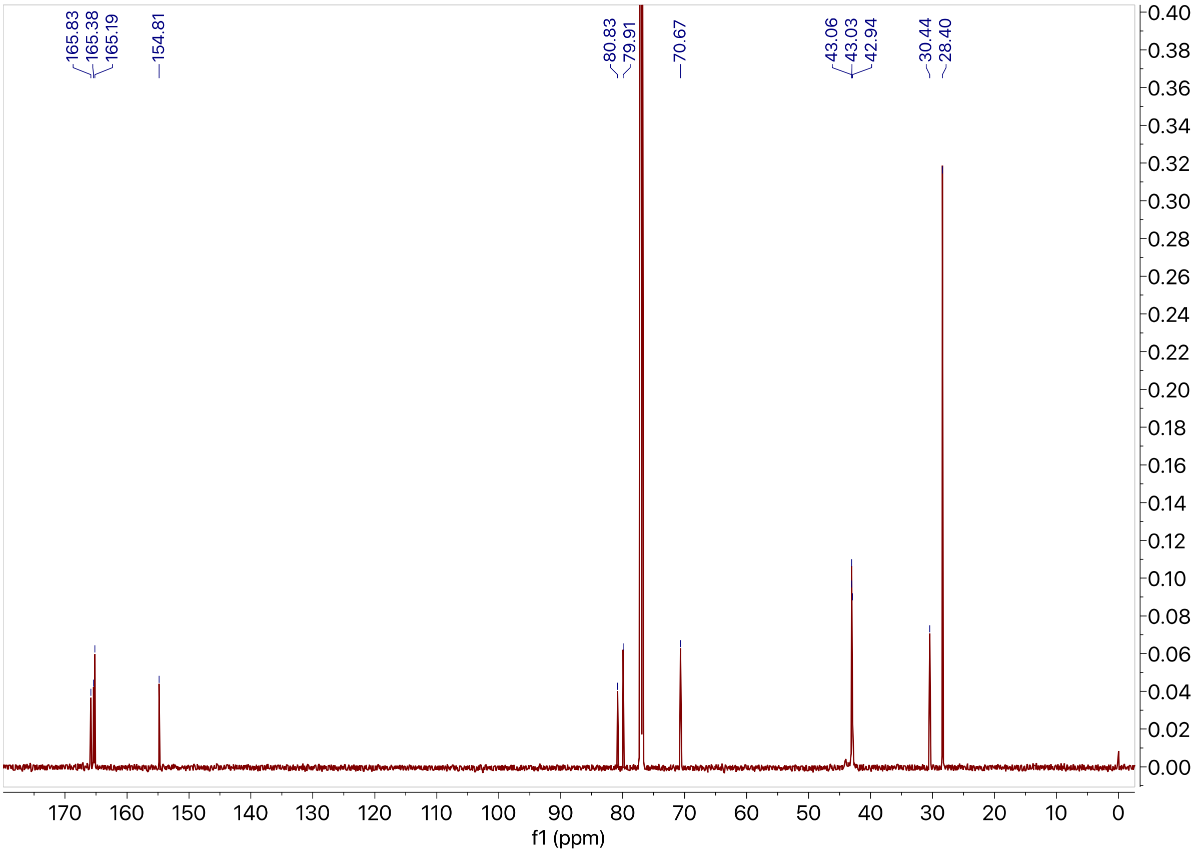

G1 dendrimer **4**

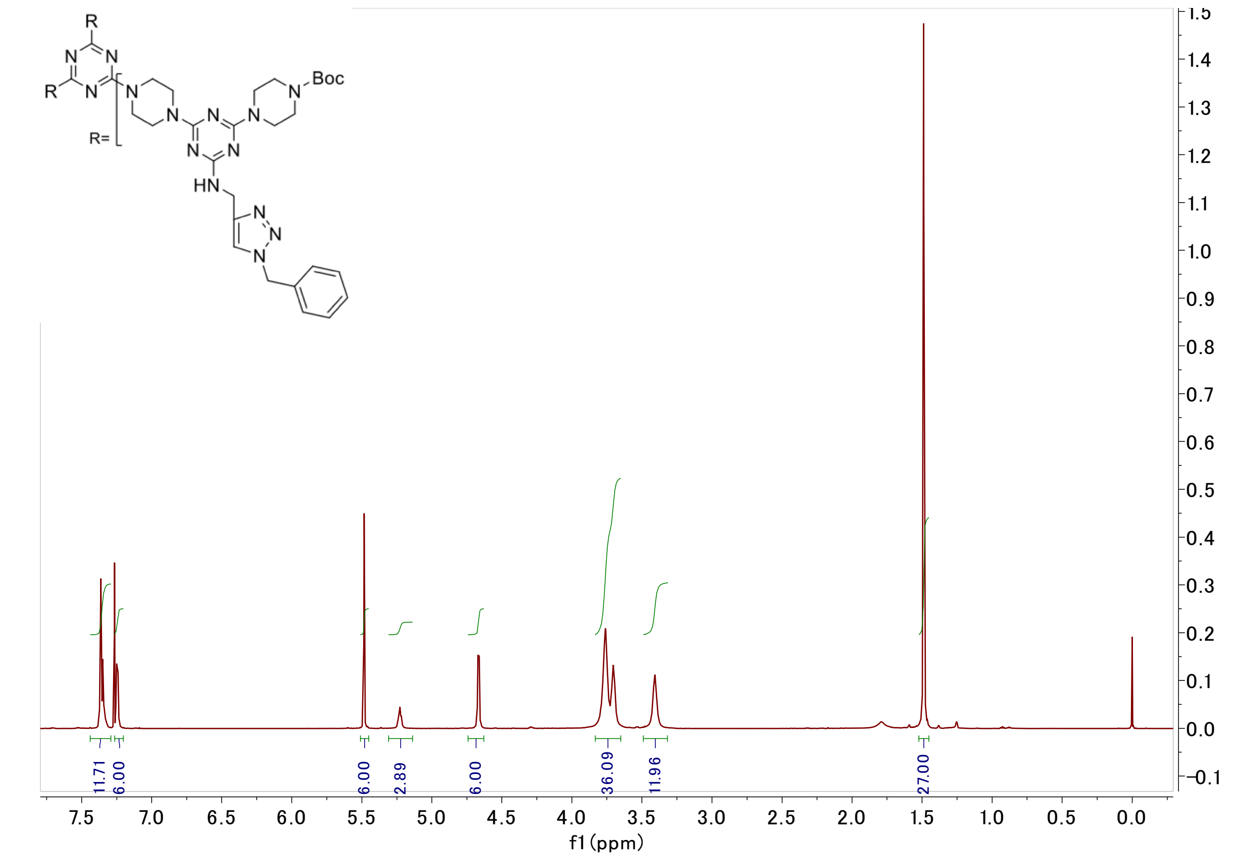

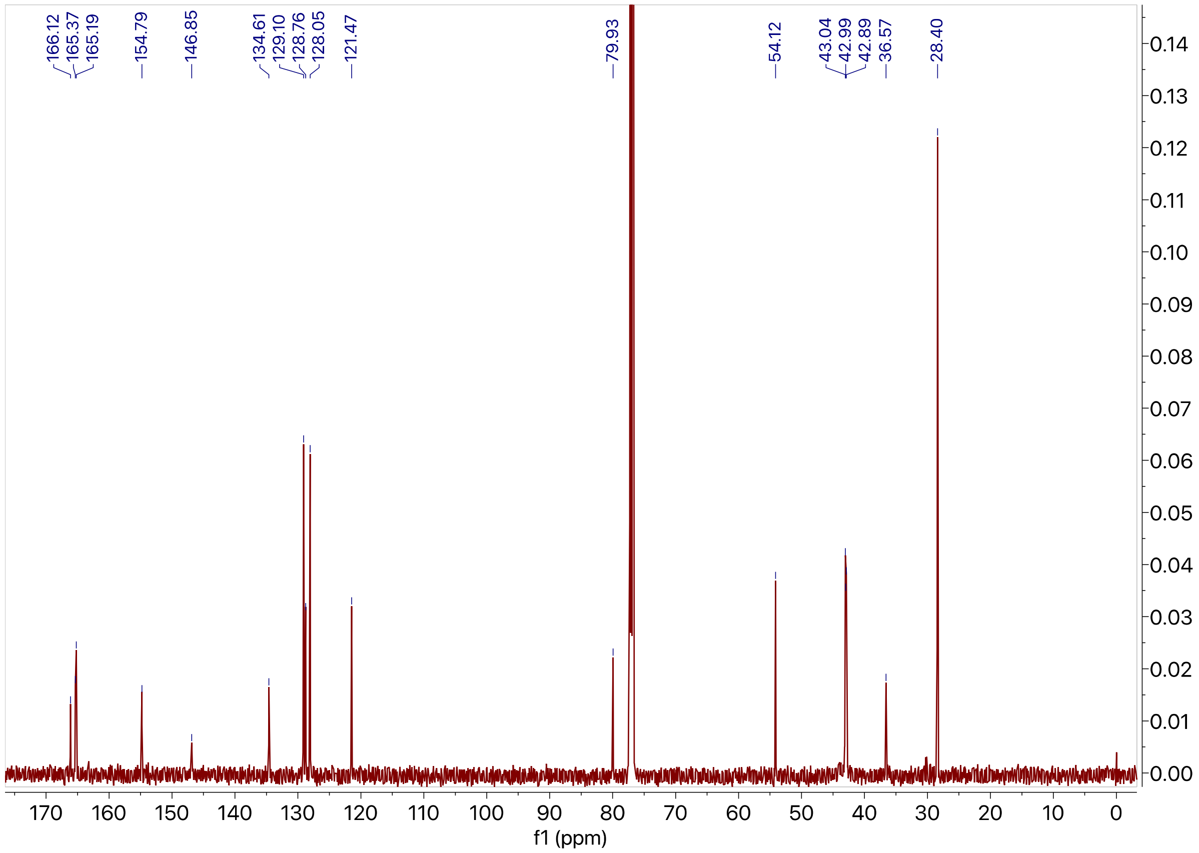

G1 dendrimer **5**

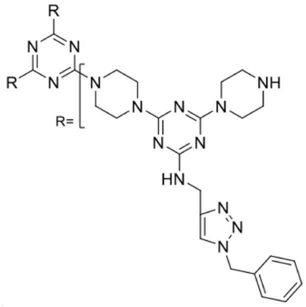

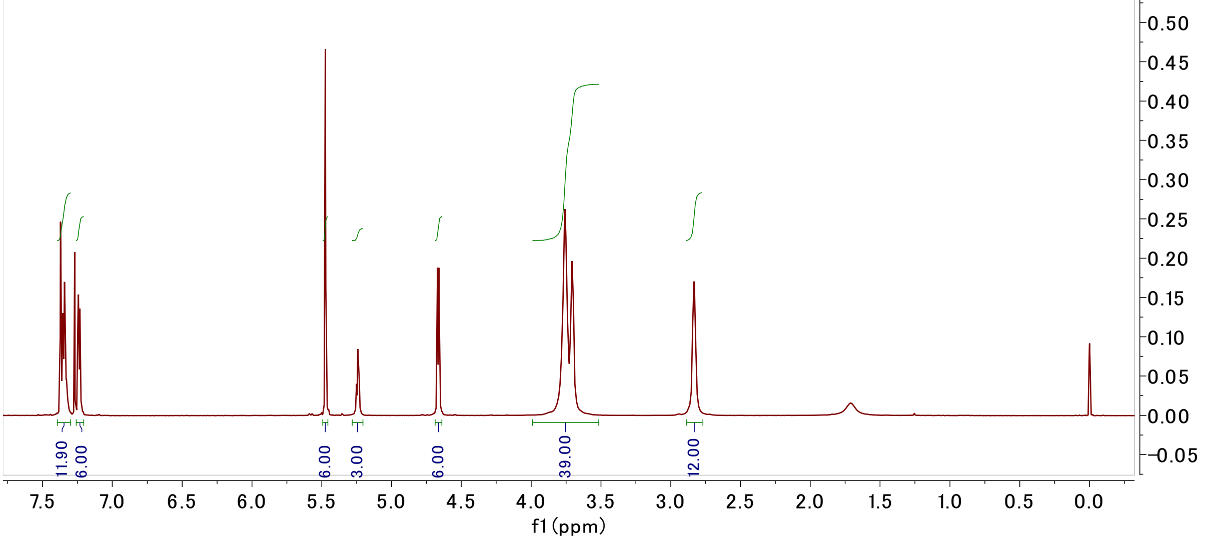

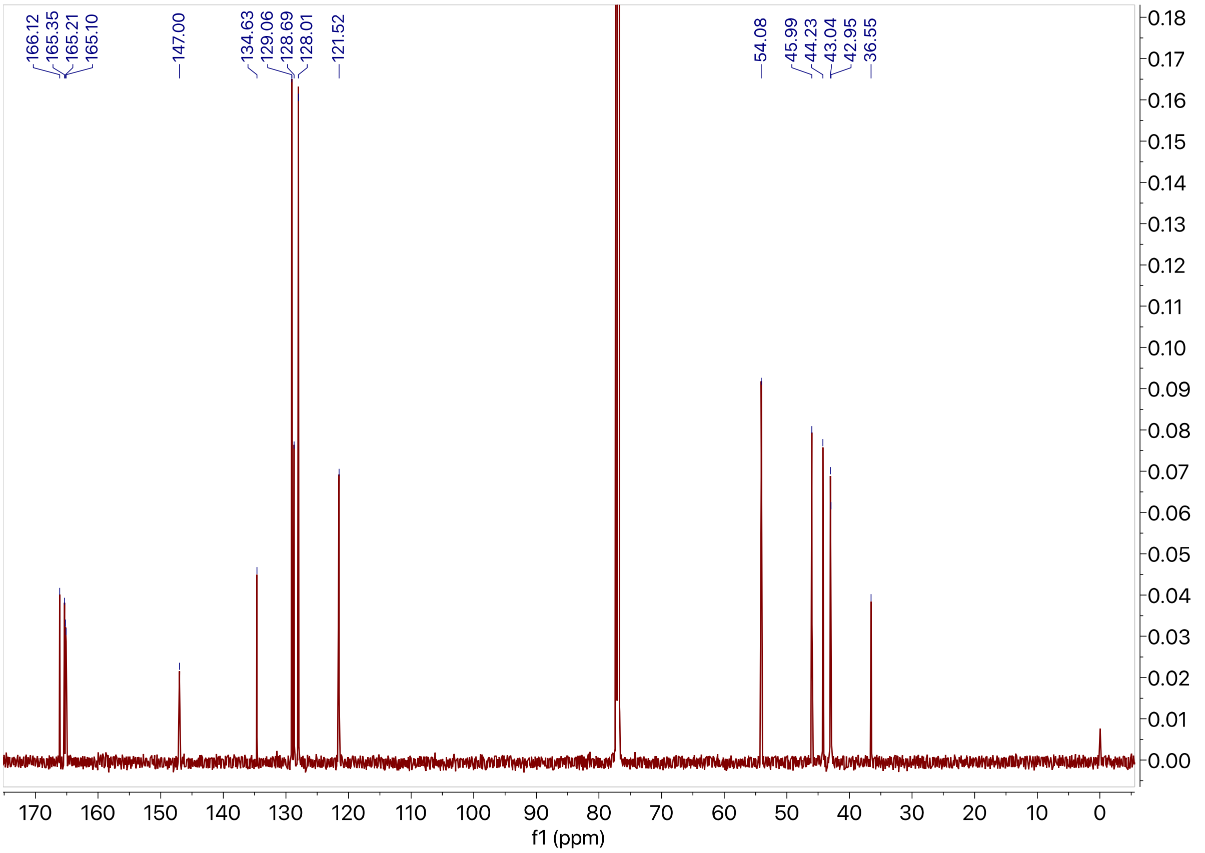

G2 dendrimer **6**

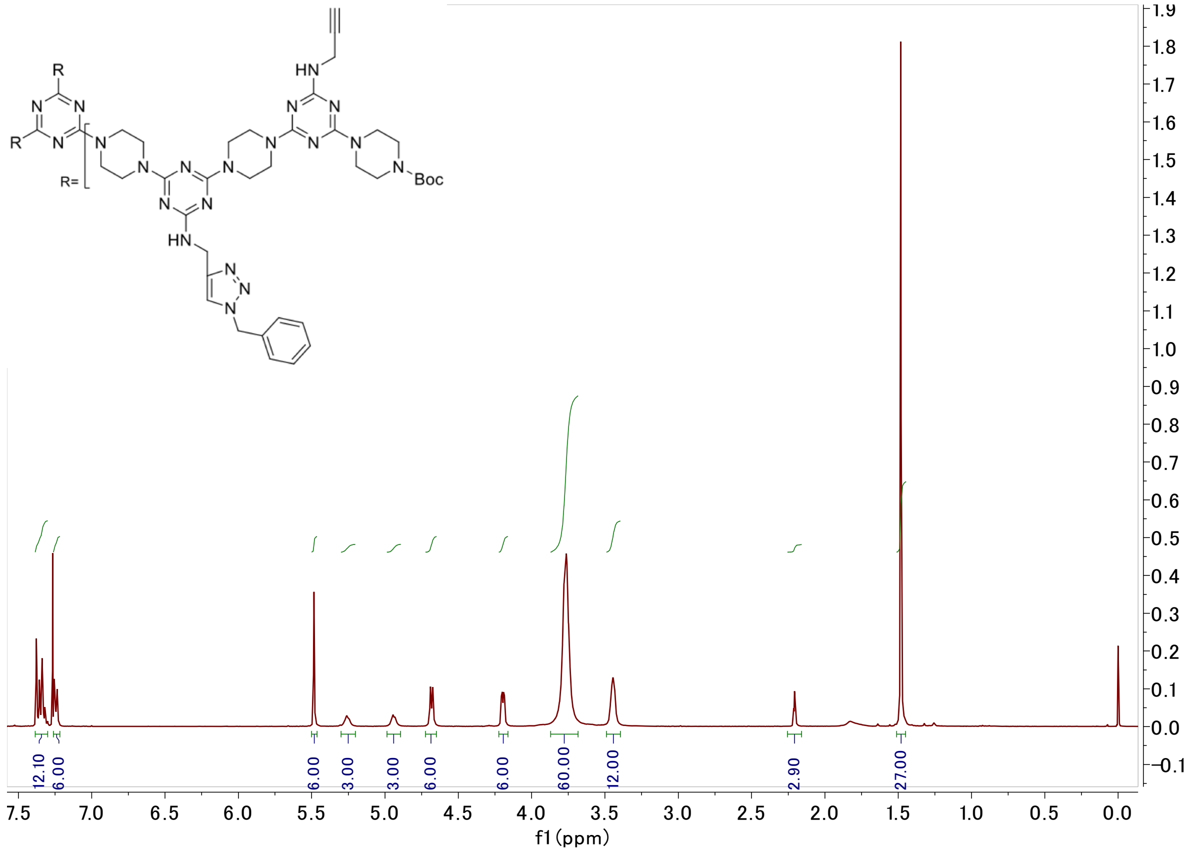

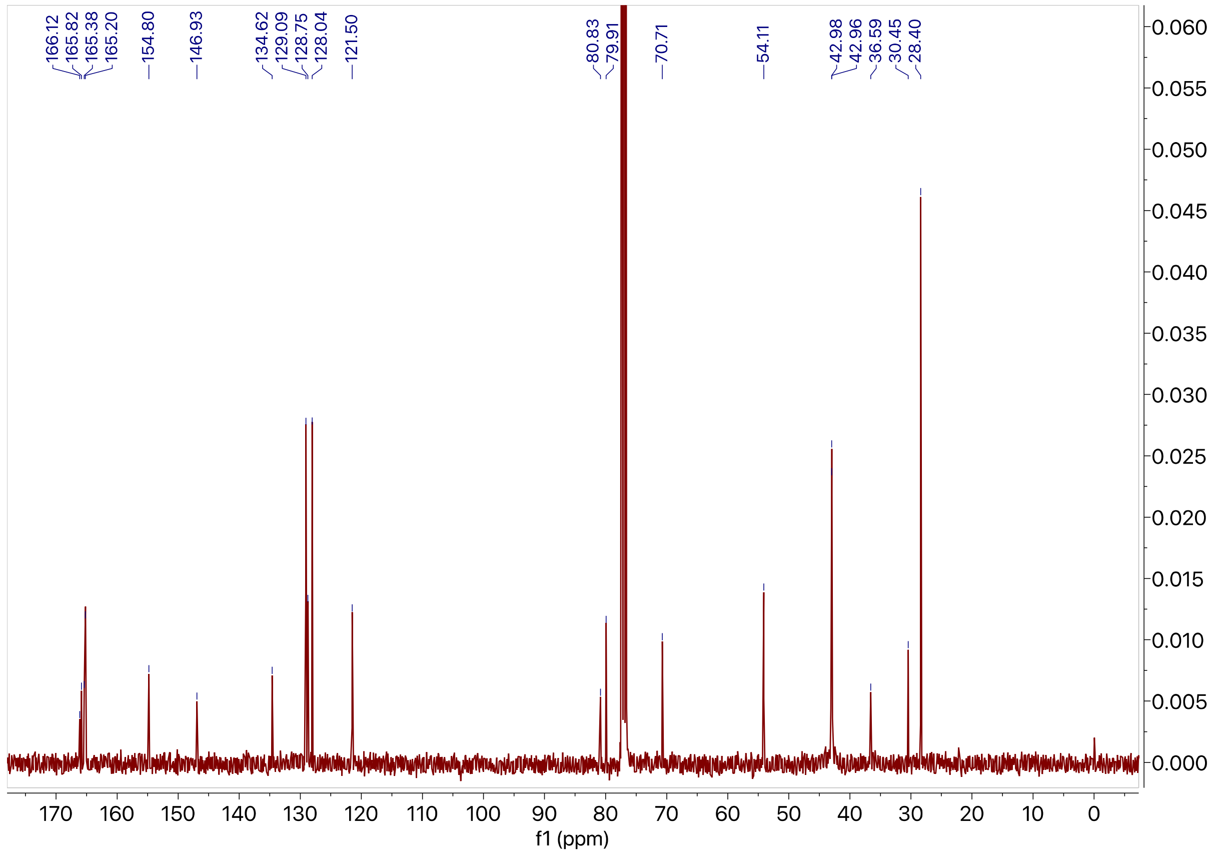

G2 dendrimer **7**

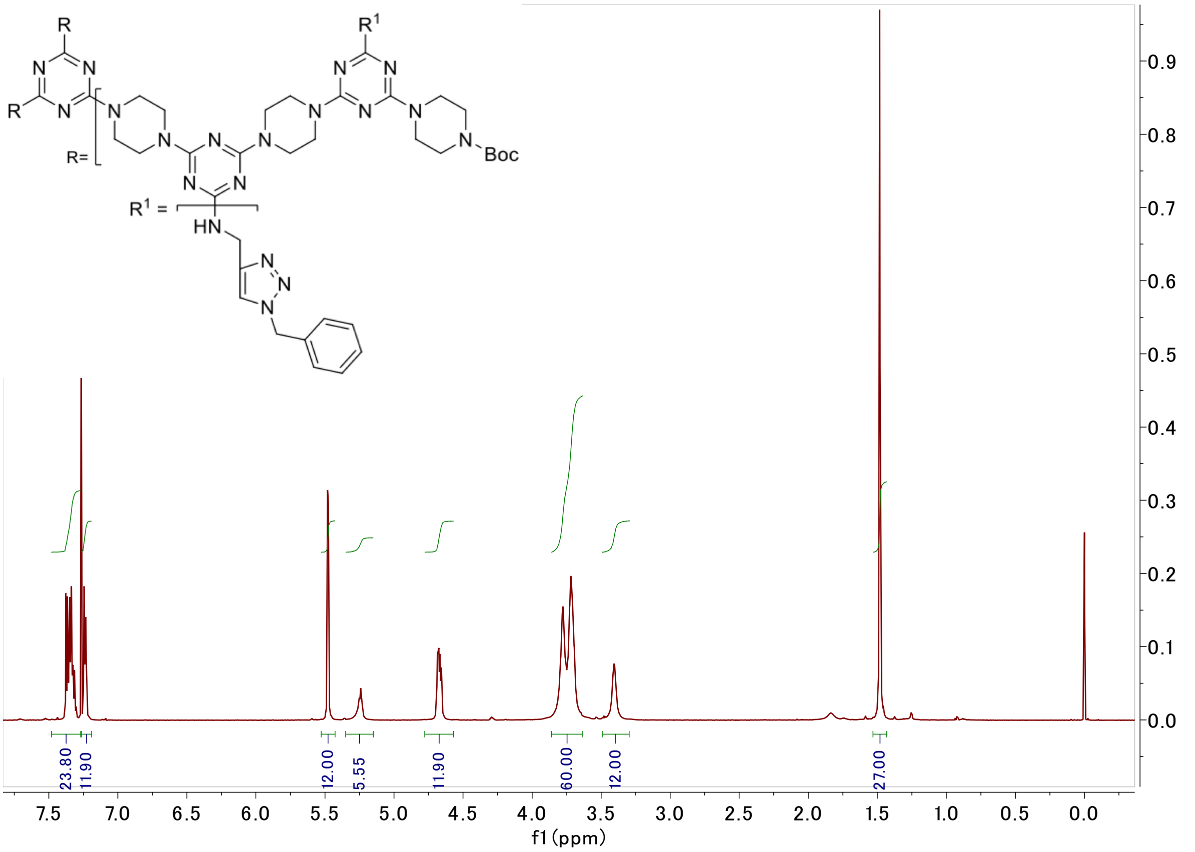

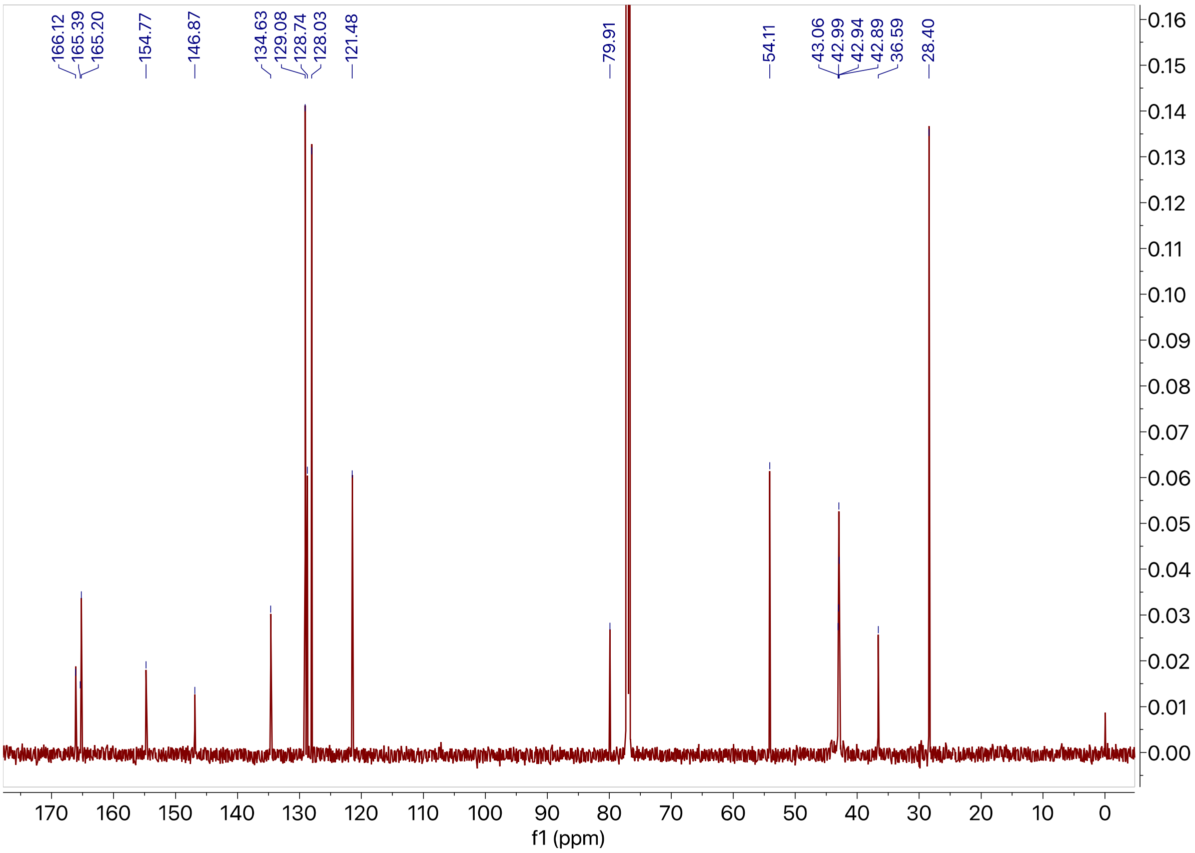

G2 dendrimer **8**

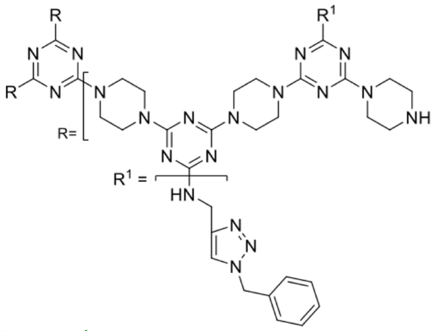

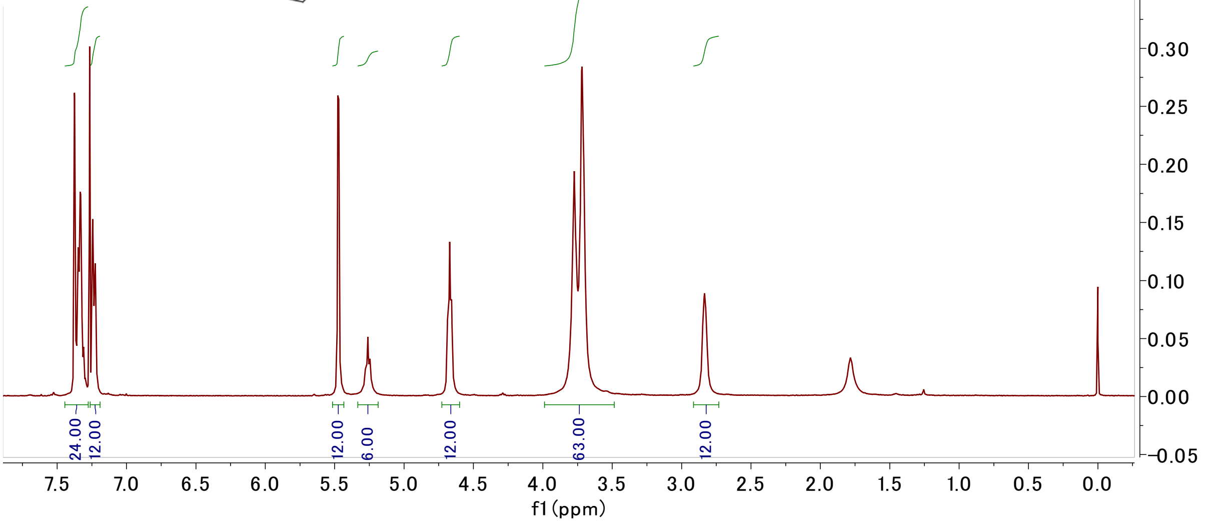

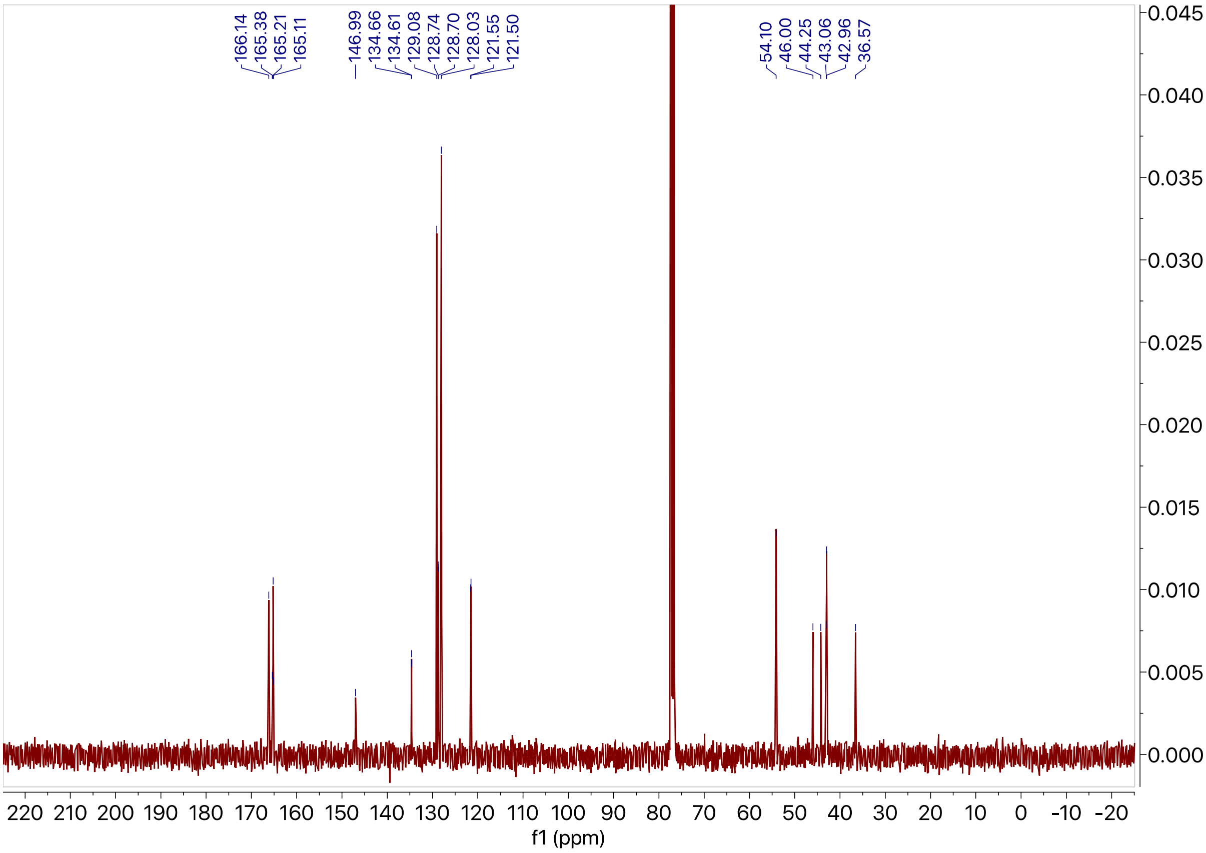

G3 dendrimer **9**

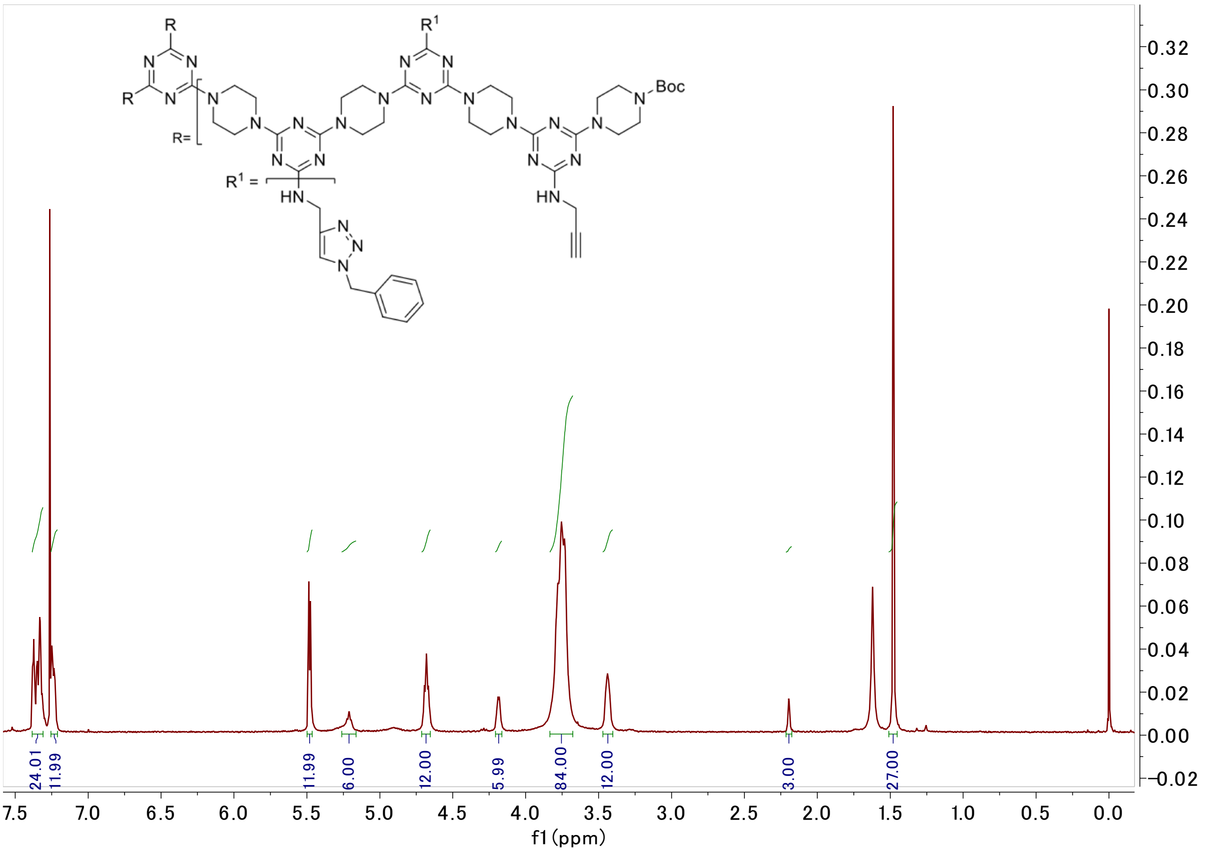

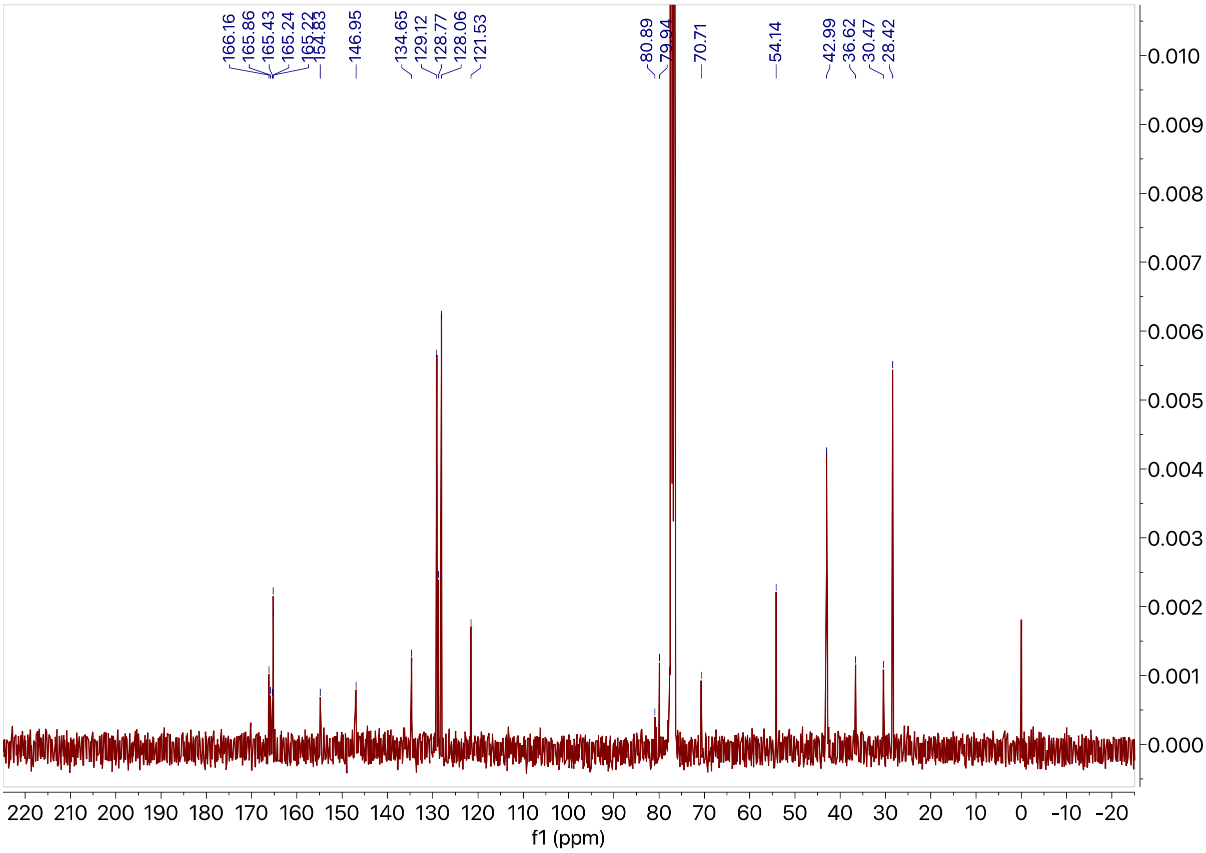

G3 dendrimer **10**

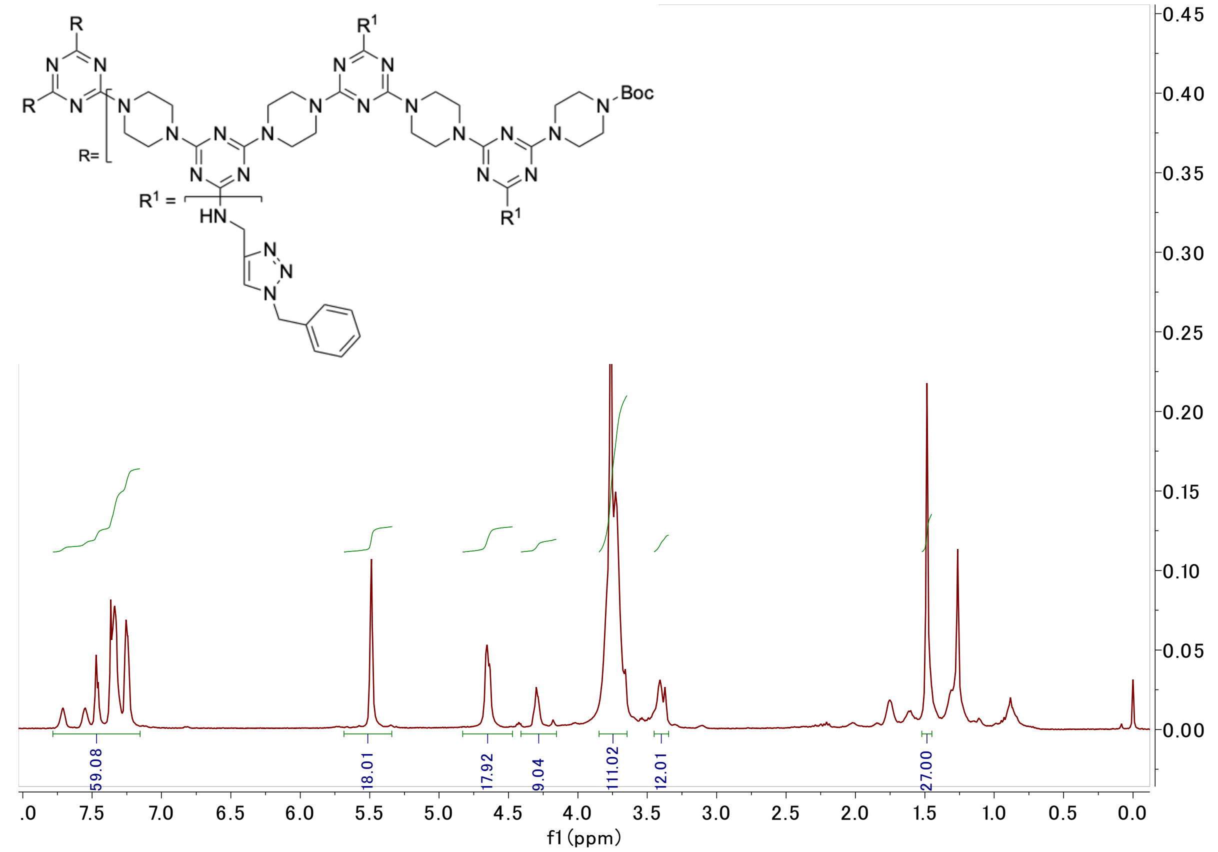

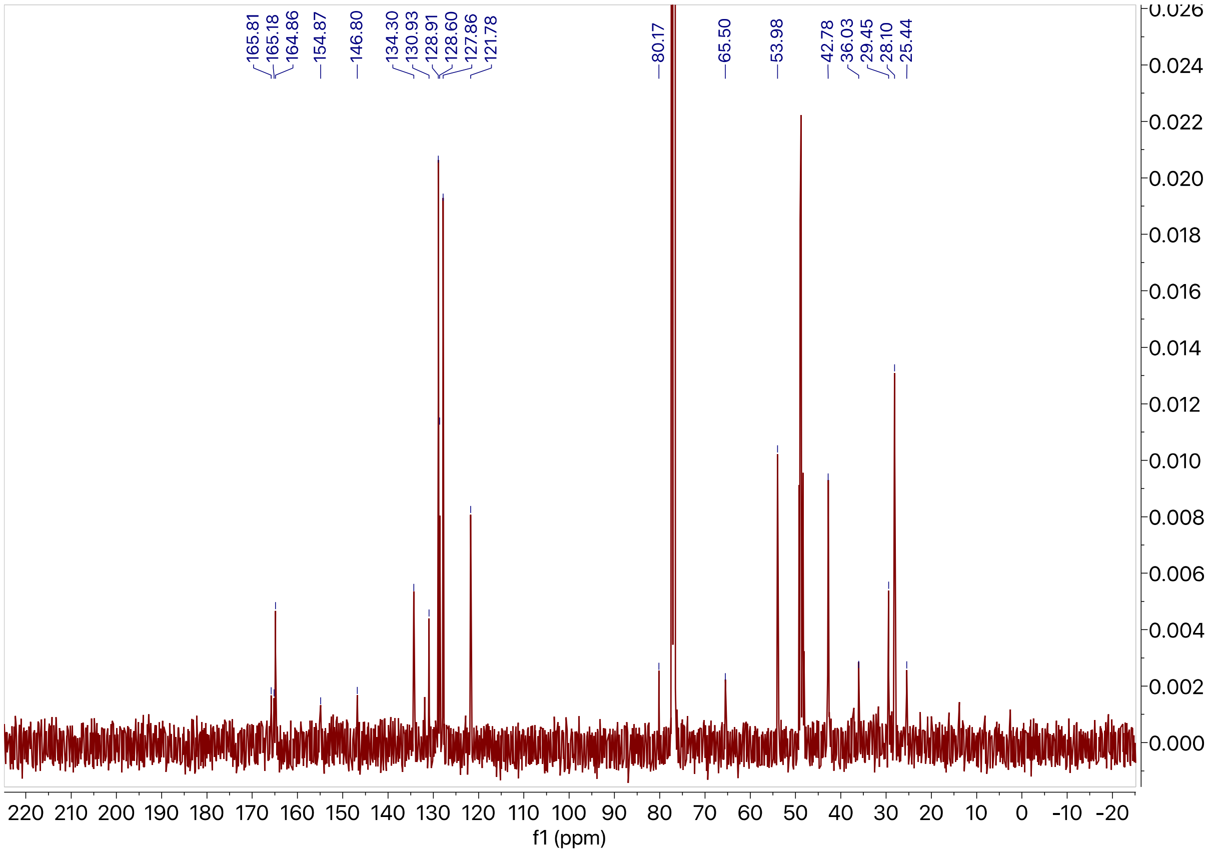

G1 dendrimer **11**

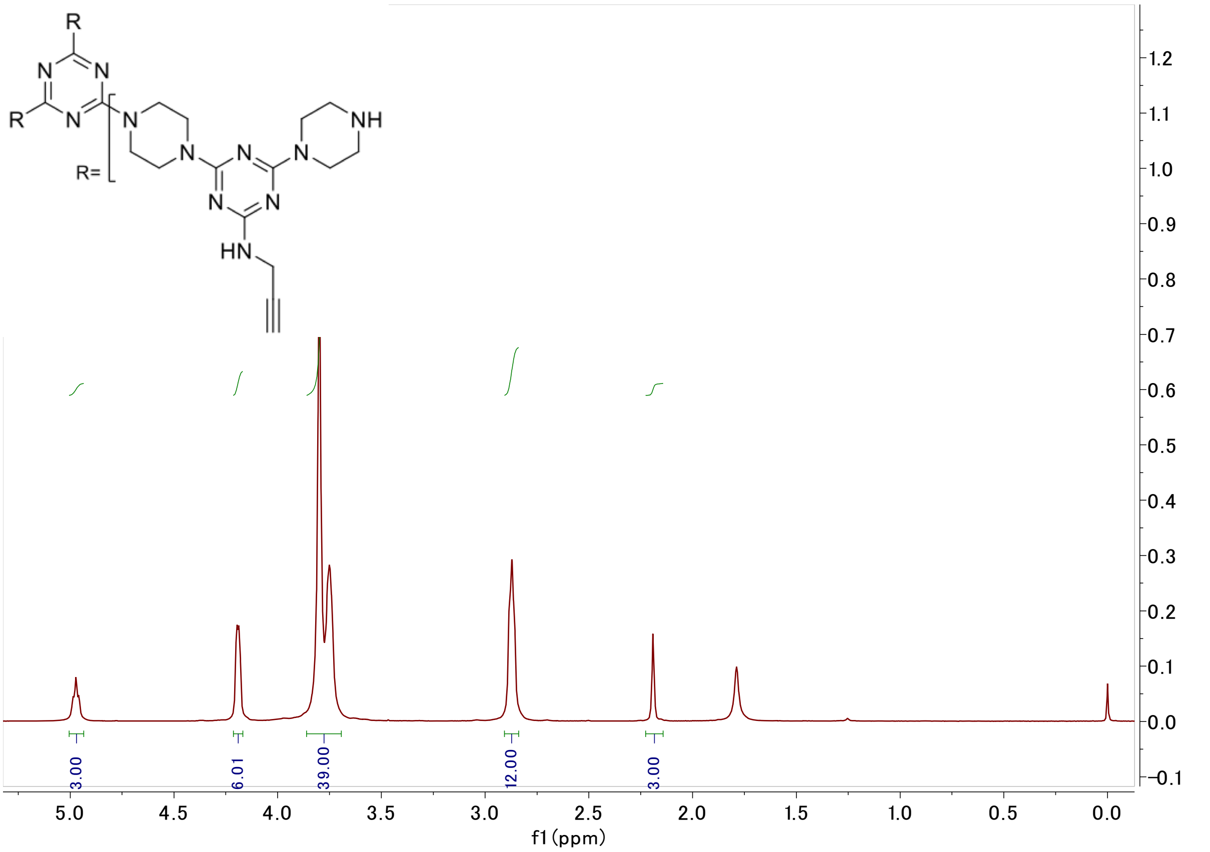

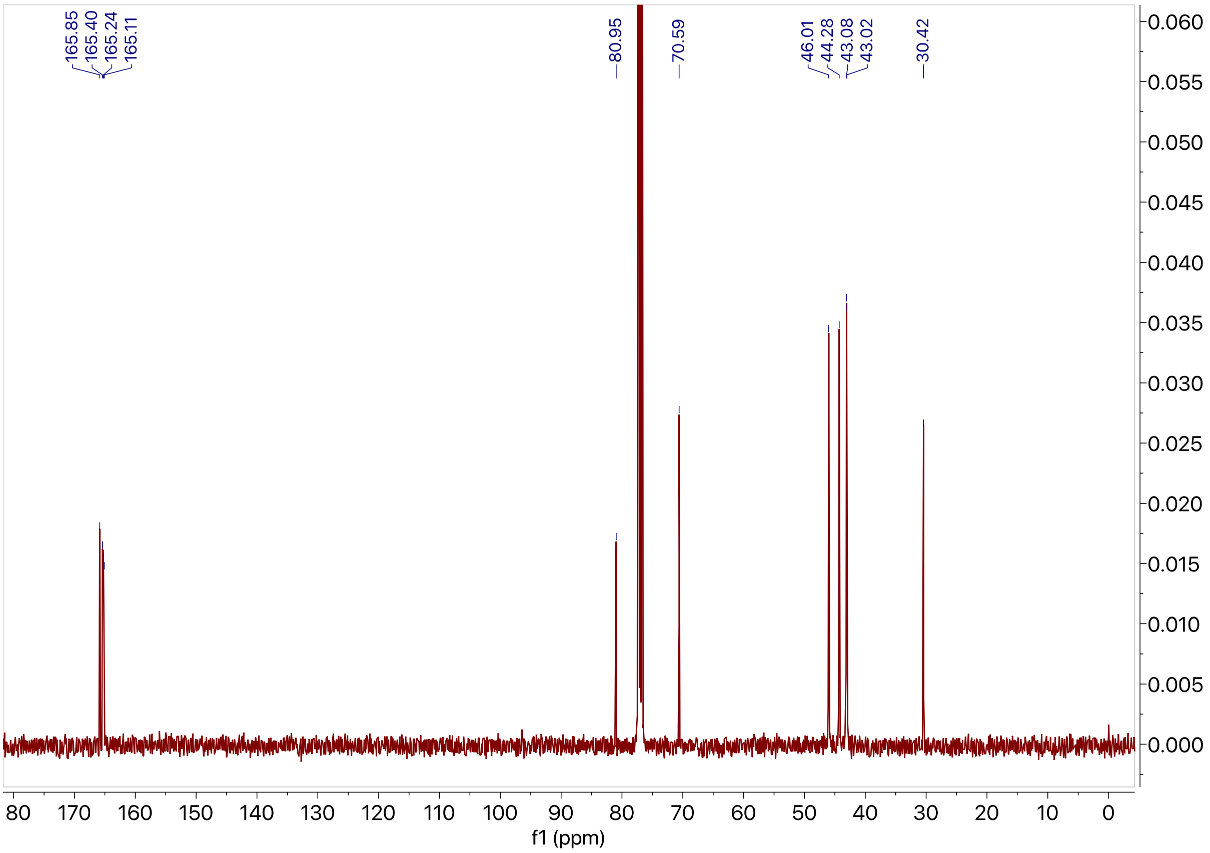

G2 dendrimer **12**

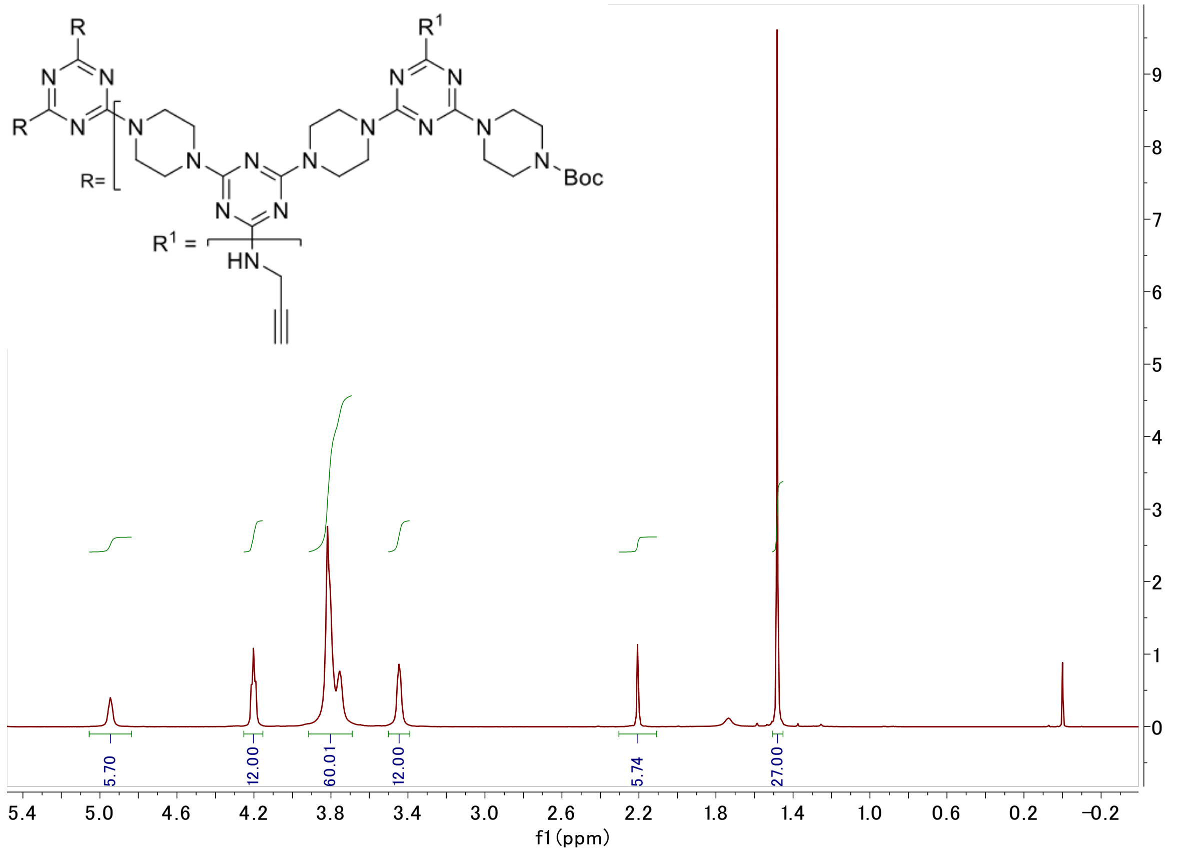

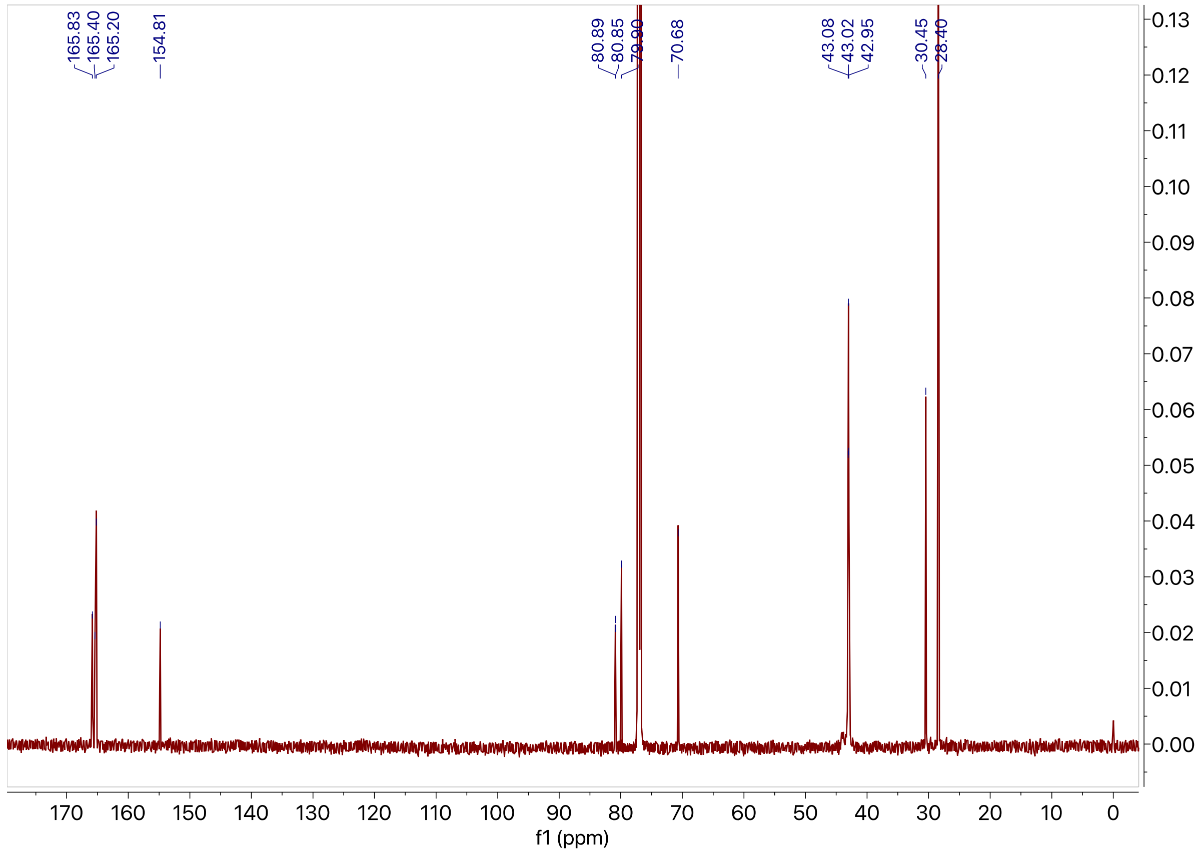

G2 dendrimer **13**

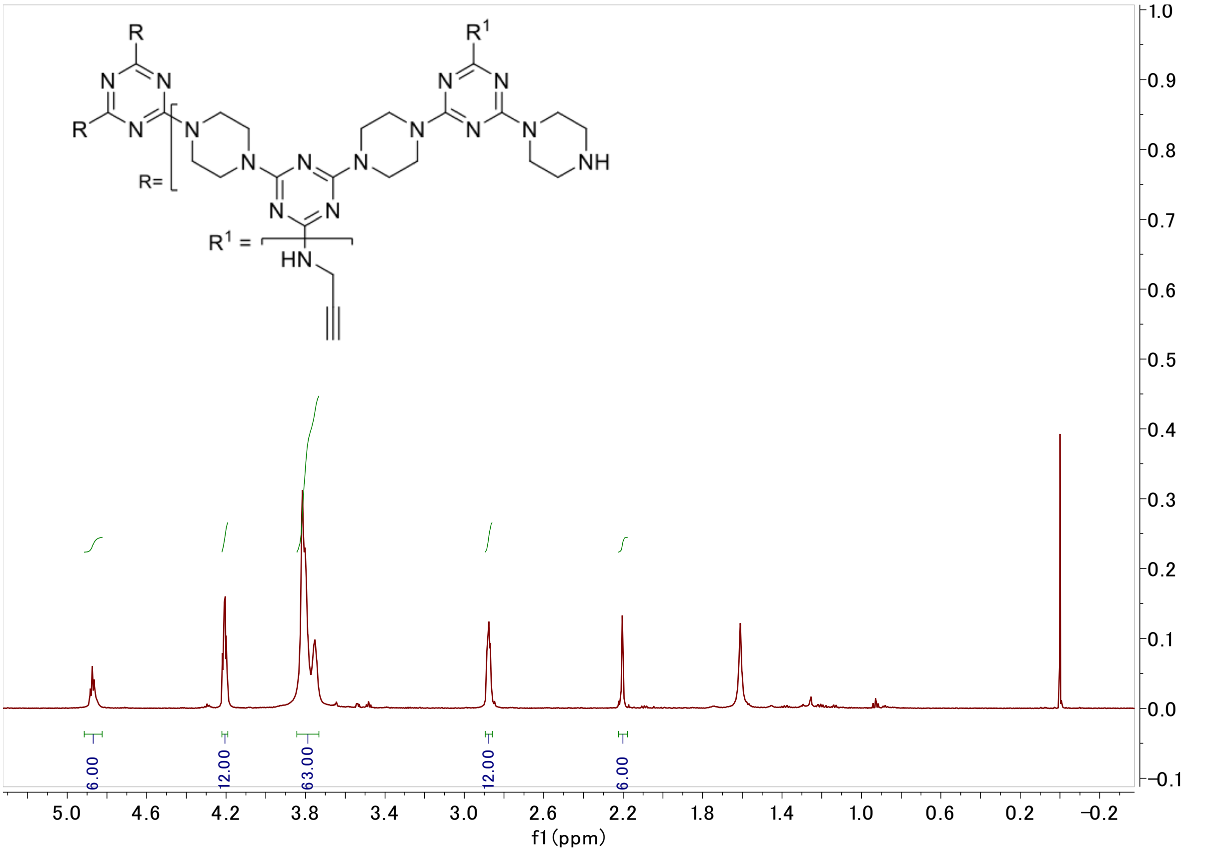

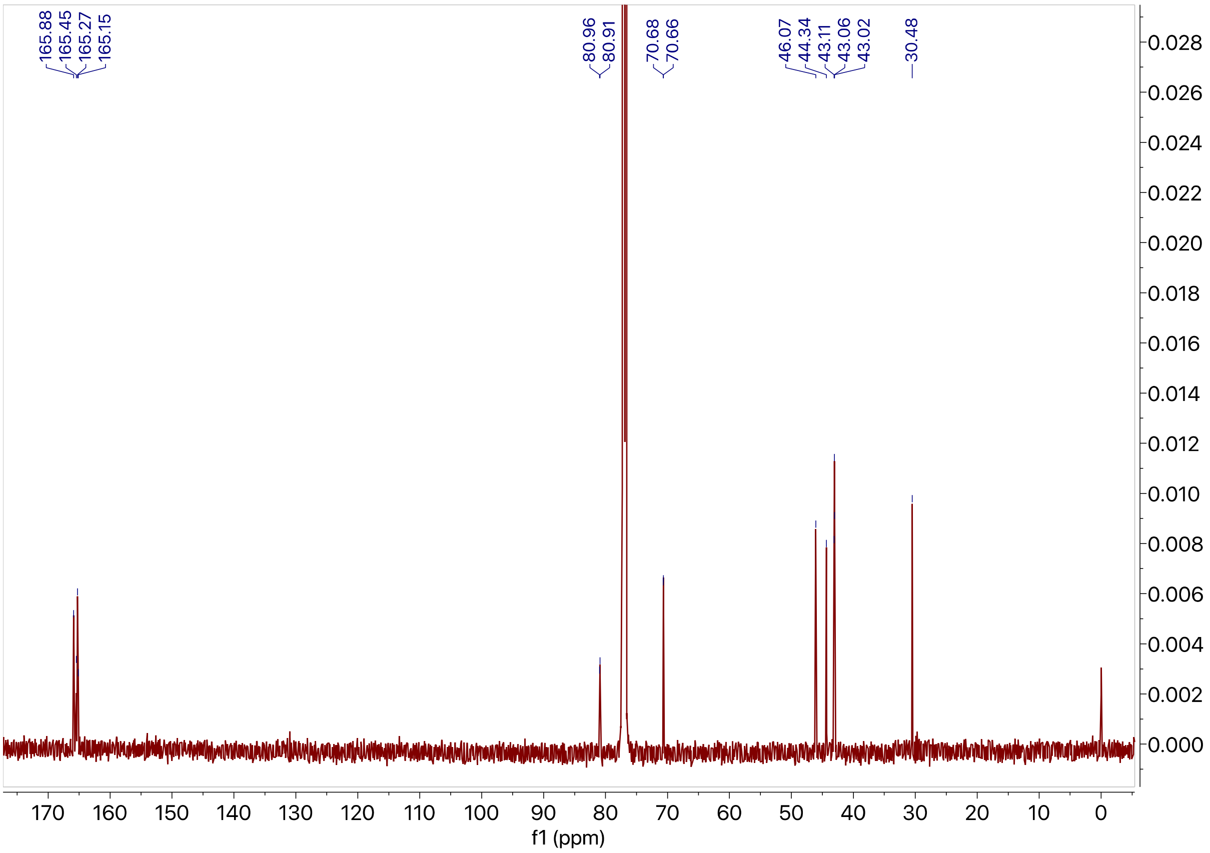

Compound **15**

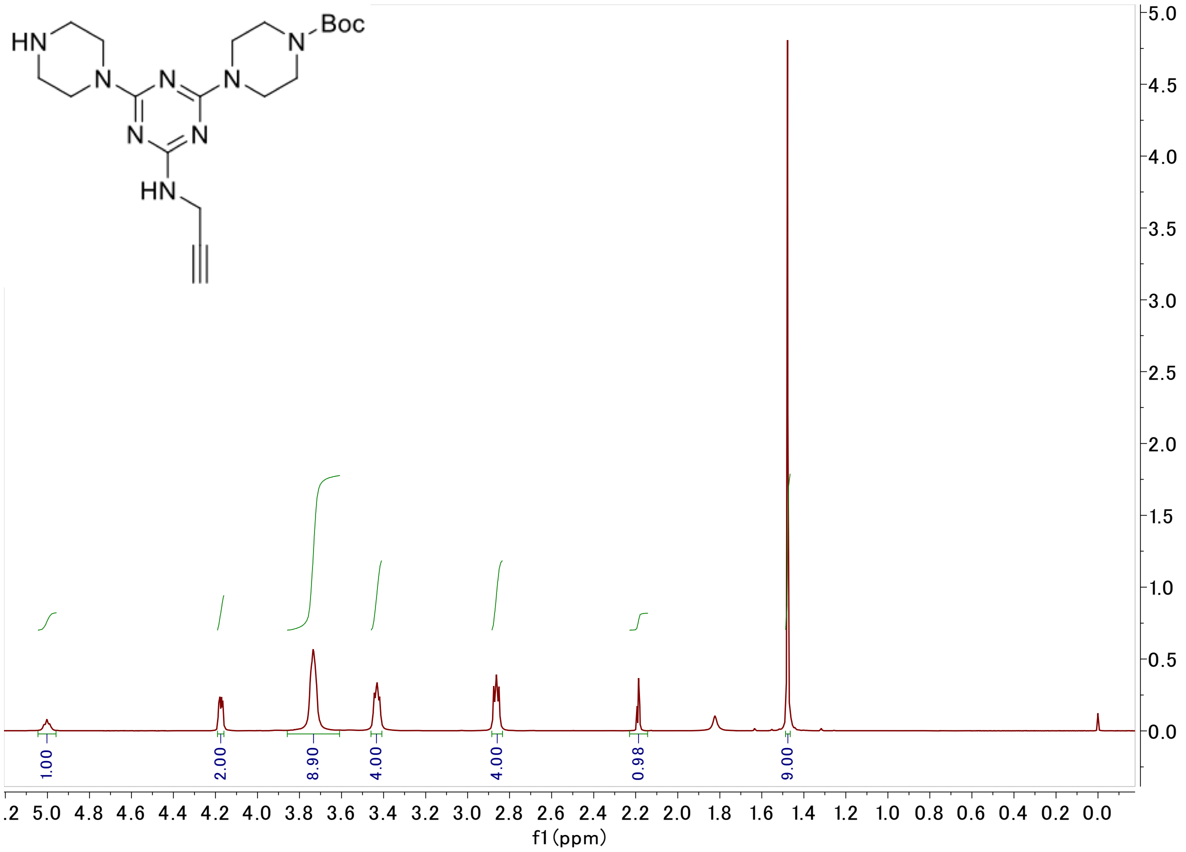
Boc
HN
N
HN
1.00
2.00
8.90
4.00
4.00
0.98
9.00
f1 (ppm)

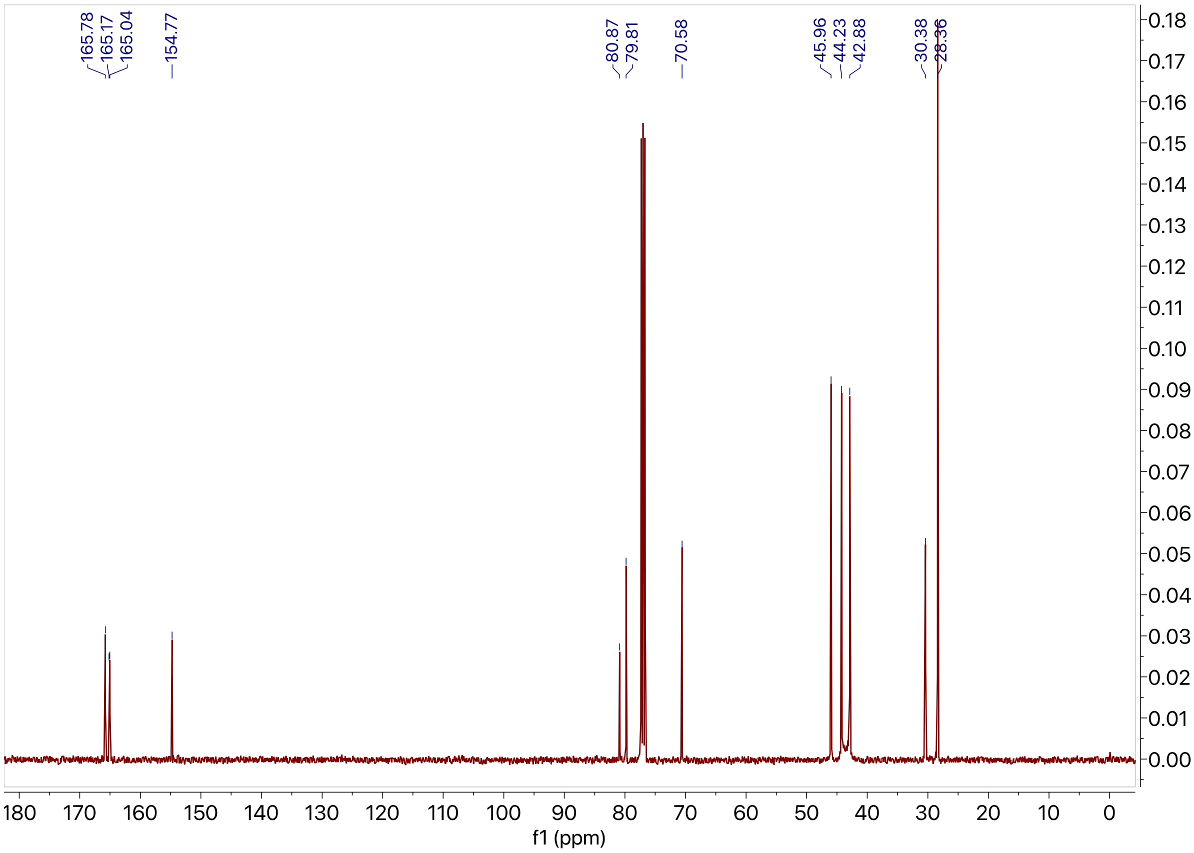
165.78
165.17
165.04
154.77
80.87
79.81
70.58
45.96
44.23
42.88
30.38
28.36
f1 (ppm)

Compound **16**

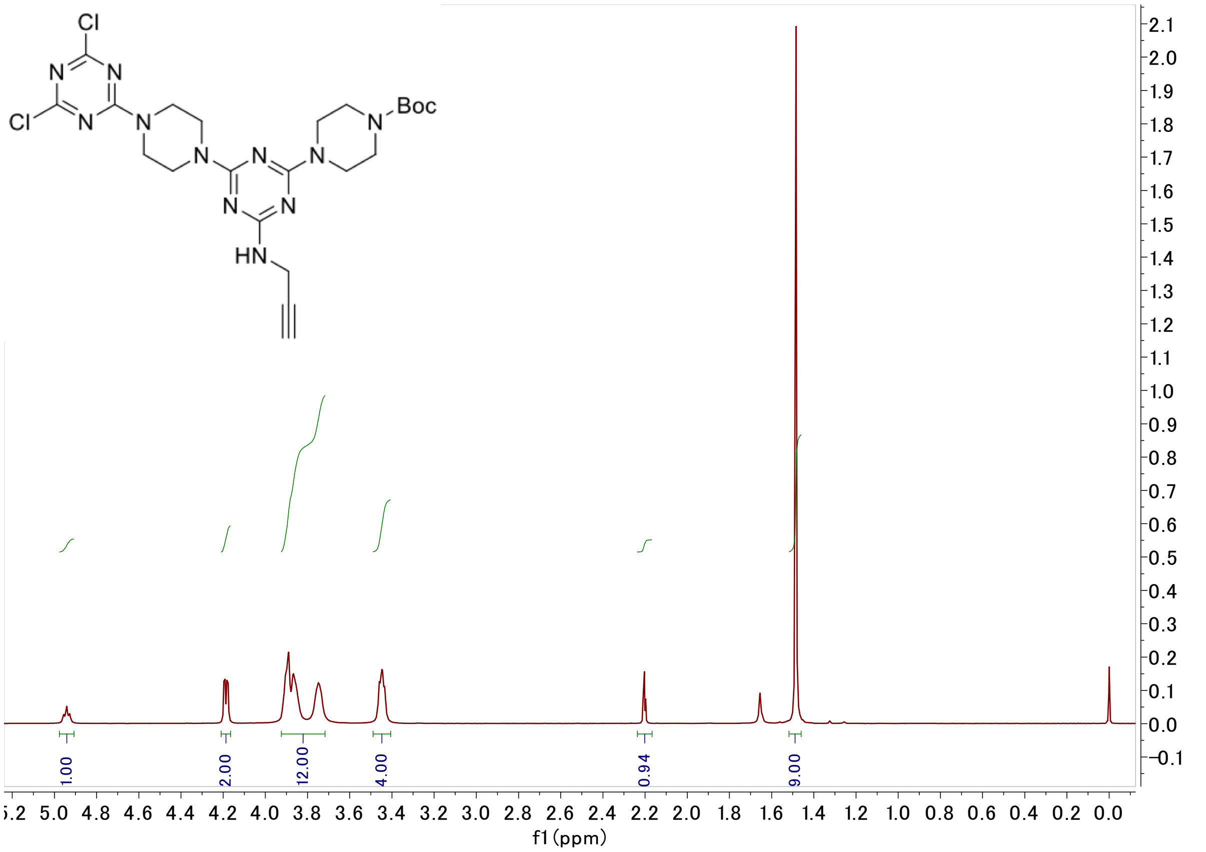
Cl
N
N
Cl
N
N
N
N
N
N
N
Boc
HN
1.00
2.00
12.00
4.00
0.94
9.00
f1 (ppm)

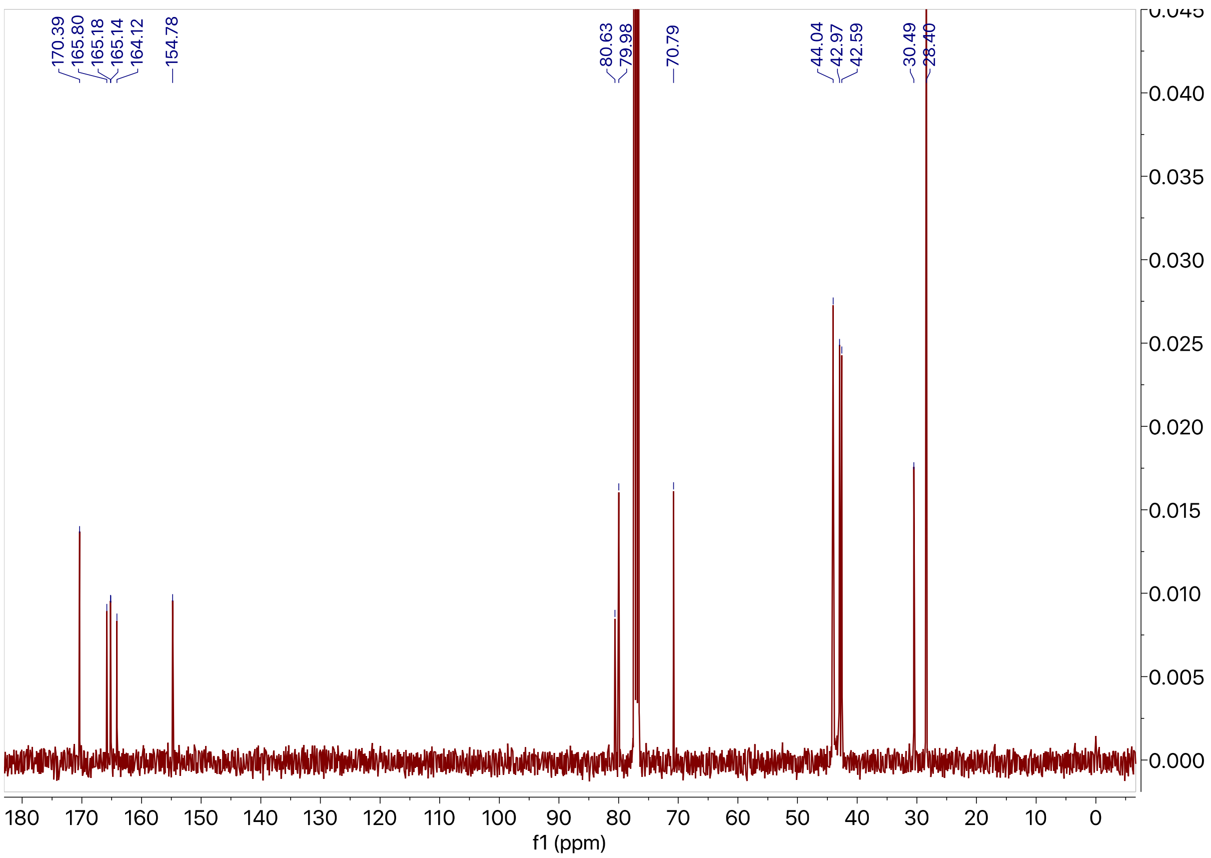
170.39
165.80
165.18
165.14
164.12
154.78
80.63
79.98
70.79
44.04
42.97
42.59
30.49
28.40
f1 (ppm)

Compound **17**

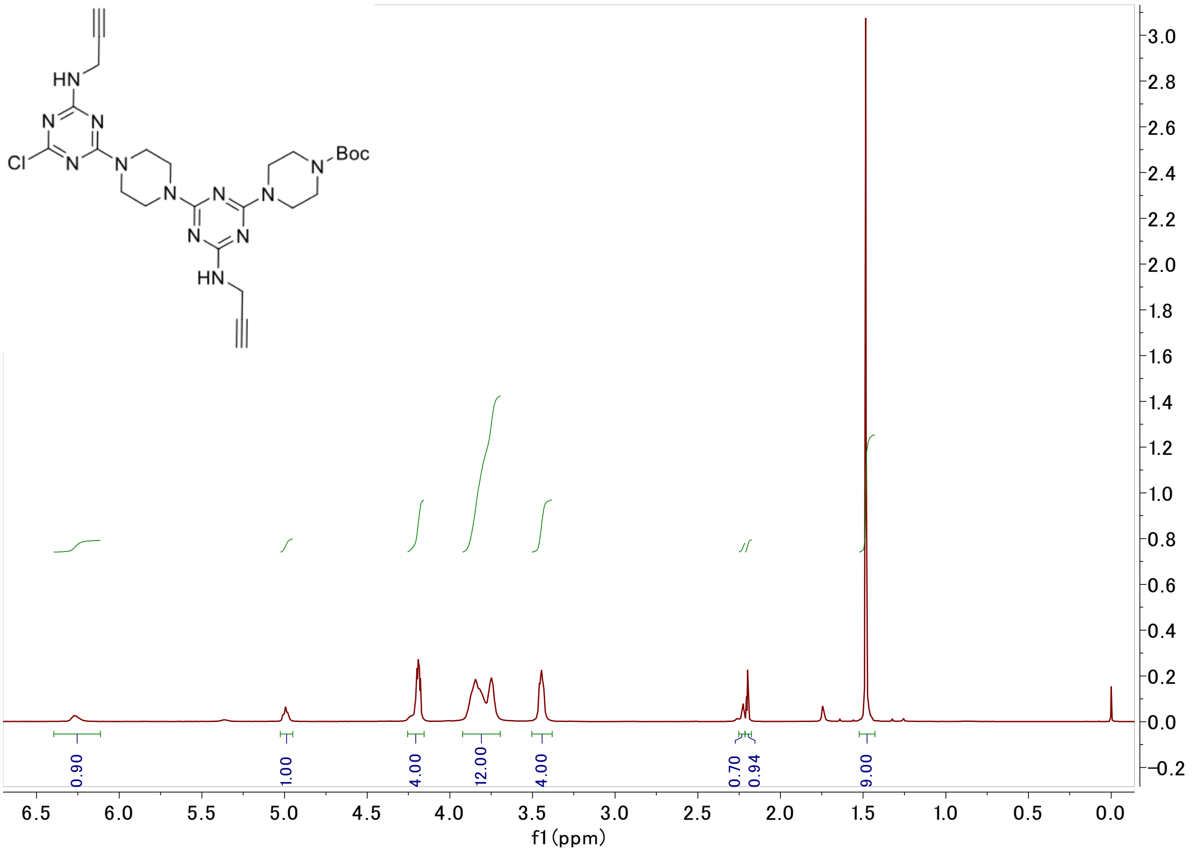
HN
Cl
Boc
0.90
1.00
4.00
12.00
4.00
0.70
0.94
9.00
f1 (ppm)

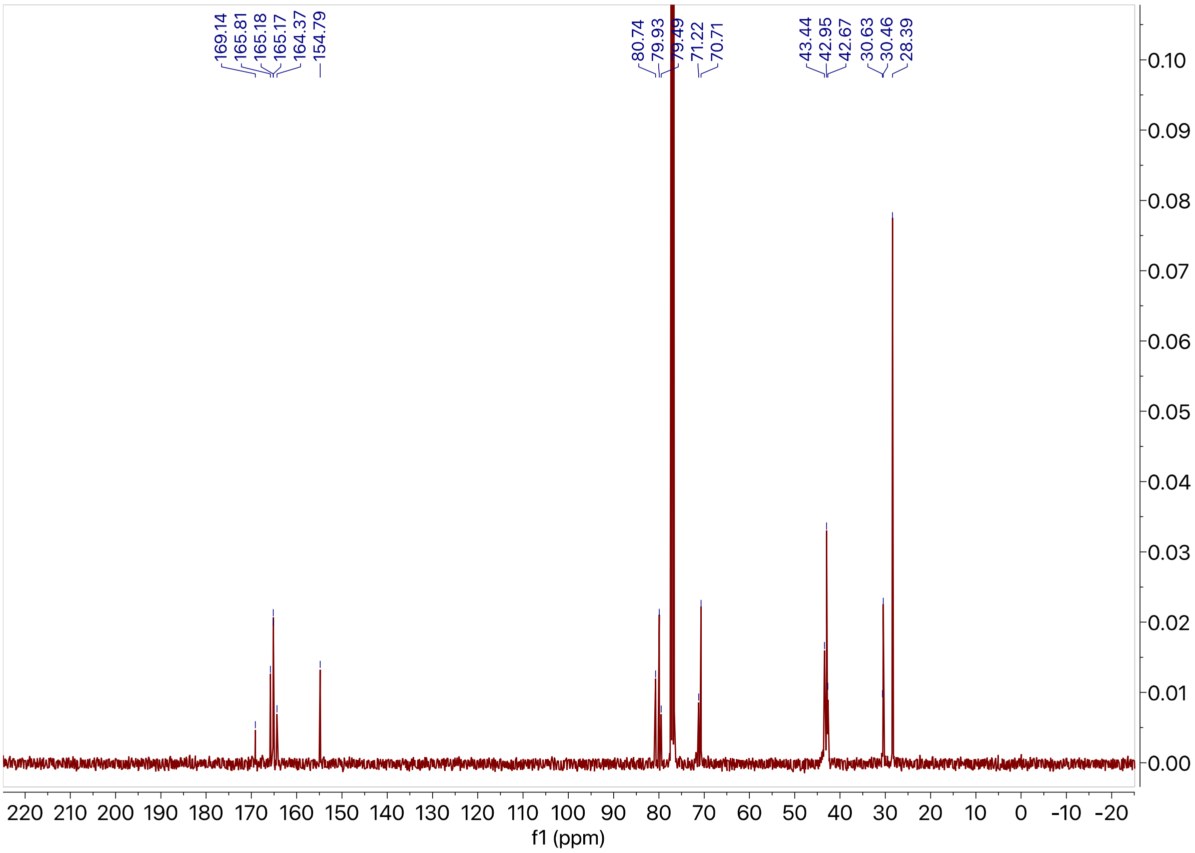
169.14
165.81
165.18
165.17
164.37
154.79
80.74
79.93
79.49
71.22
70.71
43.44
42.95
42.67
30.63
30.46
28.39
f1 (ppm)

G3 dendrimer **18**

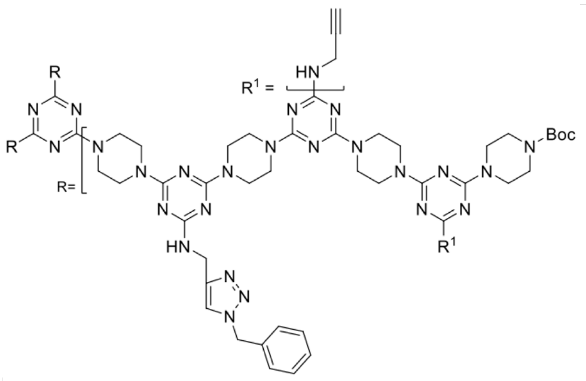

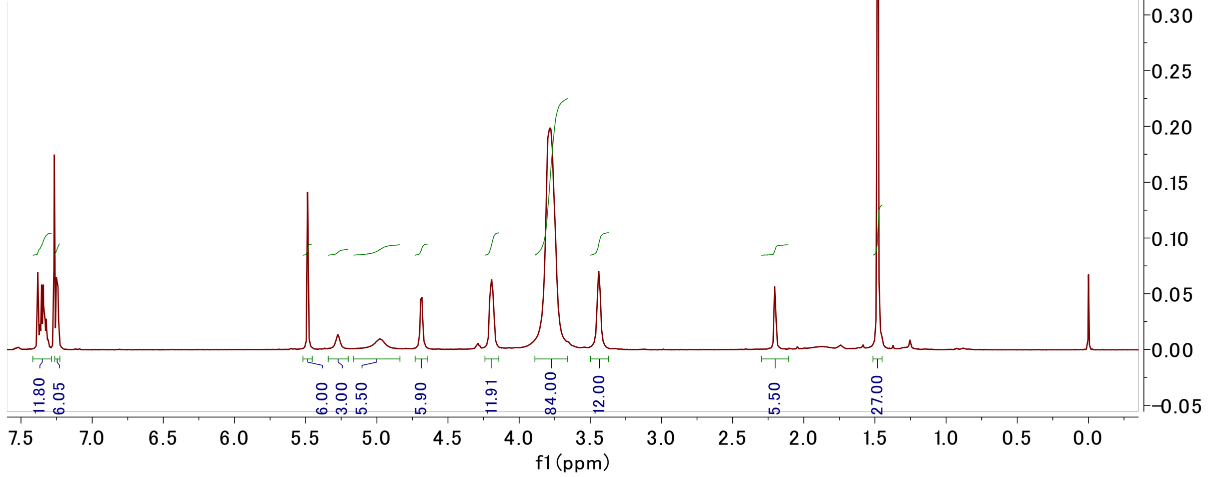

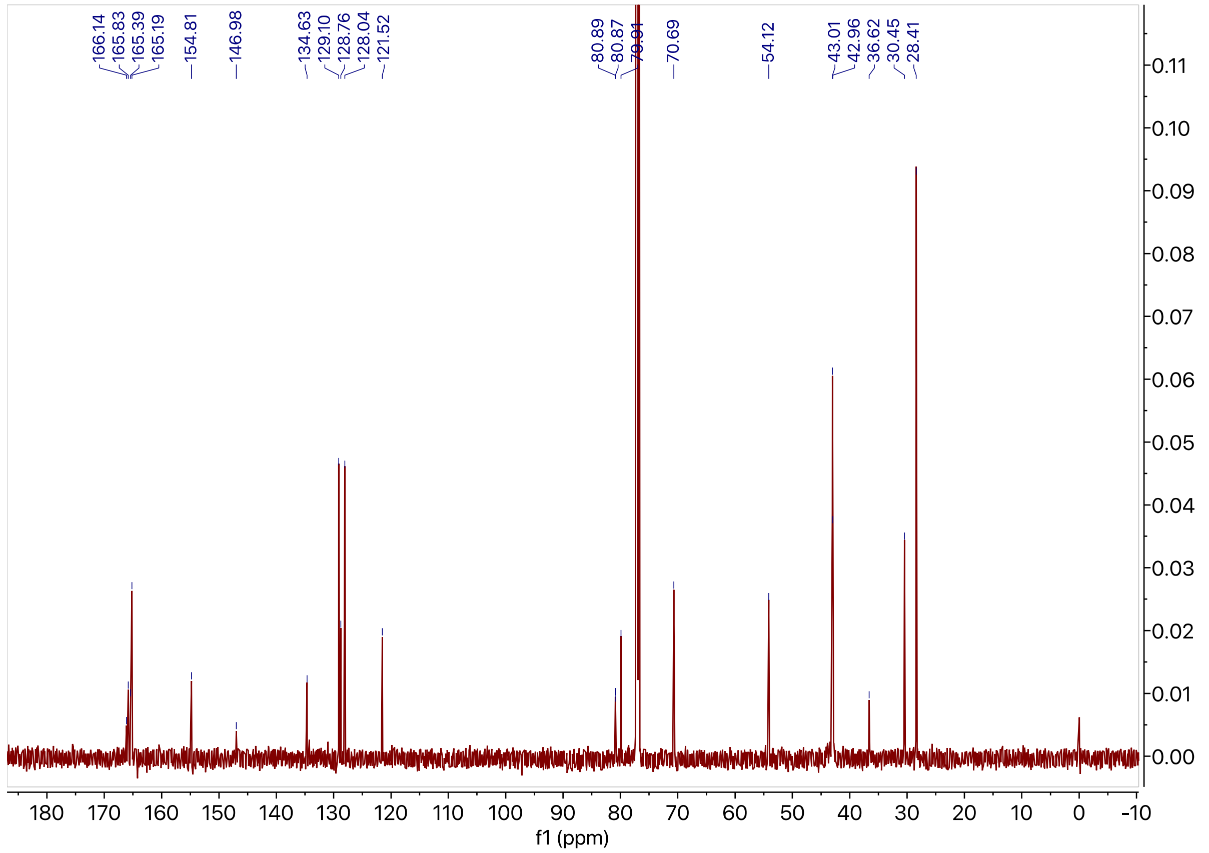

Supplement: Supplementary file 1 [file molecules-28-00131-s001.zip › molecules-2112283-supplementary.pdf]
